# Diflufenican Perturbation Reshapes Bacterial Community Structure and Alters Functional Potential Across Agricultural Soil Depths

**DOI:** 10.3390/microorganisms14071531

**Published:** 2026-07-13

**Authors:** Wenbo Wang, Pan Wang, Yuning Wei, Lixing Ding, Siqi Liu, Hale Yu, Zhaodi Wang, Fengshan Yang, Haiyan Fu

**Affiliations:** Engineering Research Center of Agricultural Microbiology Technology, Ministry of Education, Heilongjiang Provincial Key Laboratory of Ecological Restoration and Resource Utilization for Cold Region, Key Laboratory of Molecular Biology, College of Heilongjiang Province, School of Life Sciences, Heilongjiang University, Harbin 150080, China; 15729281421@163.com (W.W.); wpan222@163.com (P.W.); 15024950787@163.com (Y.W.); lukizi@163.com (L.D.); 2231732@s.hlju.edu.cn (S.L.); 2231735@s.hlju.edu.cn (H.Y.); 15246706629@163.com (Z.W.)

**Keywords:** diflufenican, herbicide disturbance, soil vertical depth, bacterial community structure, nutrient cycling

## Abstract

Diflufenican is a herbicide widely used in agricultural systems, but its effects on soil bacterial communities across different soil depths remain insufficiently understood. In this study, we investigated depth-resolved bacterial community responses and functional potential following diflufenican perturbation in agricultural soils. Soil samples collected from different depths were incubated with or without diflufenican, and bacterial communities were analyzed using 16S rRNA gene amplicon sequencing. Co-occurrence network analysis was conducted to explore potential bacterial association patterns, and PICRUSt2 and FAPROTAX were used to infer bacterial functional potential. Diflufenican perturbation altered bacterial alpha diversity and reshaped community composition, with response patterns varying among soil depths. Principal coordinate analysis based on Bray–Curtis dissimilarity showed separation between control and diflufenican-treated soils, indicating treatment-associated shifts in bacterial community structure. Several genera, including *Sphingomonas*, *Pseudarthrobacter*, *Bacillus*, *Microvirga*, and *Phenylobacterium*, were enriched in diflufenican-treated soils, suggesting that they may represent candidate diflufenican-associated taxa. Co-occurrence network analysis further indicated changes in potential bacterial association patterns after diflufenican perturbation. Functional inference suggested shifts in bacterial functional potential, particularly in nutrient cycling-related functions, and these shifts differed among soil depths. Overall, these findings indicate that diflufenican perturbation reshapes soil bacterial community structure and alters inferred functional potential in a depth-dependent manner, highlighting the importance of soil vertical depth in assessing herbicide impacts on agricultural soil microbiomes.

## 1. Introduction

Herbicides account for 47.5% of global pesticide use. Because herbicide residues can penetrate soil profiles, potentially contaminate groundwater, and adversely affect non-target soil microorganisms, the widespread use of herbicides has created a key paradox: chemicals that ensure food production also threaten soil ecological integrity [1,2,3]. Diflufenican is a substituted pyridyloxybenzamide herbicide developed as a pre- and early post-emergence herbicide for use in cereal-based cropping systems, particularly wheat and barley, to control broadleaf weeds and certain grass weeds [4,5,6,7]. Environmentally, diflufenican is characterized by very low aqueous solubility, low volatility, strong sorption to soil organic matter, and moderate-to-high persistence, with reported half-lives ranging from 42 to 621 days under laboratory and field conditions [8,9,10]. These properties promote its retention and prolonged interaction with soil matrices following application.

However, while diflufenican’s surface soil (0–30 cm) retention is well documented [11,12], its biological consequences for bacterial communities in deeper soil horizons (30–90 cm) remain virtually unexplored. This gap is critical because microbial biomass, abundance, and activity decline markedly with depth, potentially slowing biodegradation and increasing ecological risk in subsoils [13,14,15]. There were significant differences in microbial communities between deep soil and surface soil [16,17,18,19]. In boreal forest Podzols, bacterial diversity decreases with depth, while fungal communities show strong vertical partitioning between saprotrophic-dominated organic layers and mycorrhizal-dominated mineral soils [20]. These patterns underscore that depth-specific community sensitivity to perturbation cannot be inferred from surface soil responses alone, a principle that extends to agricultural soils under herbicide stress. In agricultural soils, microbial biomass, abundance, and activity generally decrease with increasing soil depth, which can slow pesticide biodegradation and increase the spatial variability of degradation processes in subsoil environments [13,14,15]. Existing diflufenican studies have predominantly examined dissipation kinetics, persistence, and surface soil enzymatic activity, often in combination with other herbicides such as chlorotoluron, flufenacet, or mesosulfuron-methyl [10,21,22,23,24]. While herbicide-induced shifts in nitrogen-cycling functions—such as denitrification enhancement and nitrification suppression—have been reported for other compounds [25,26,27,28], the depth-specific community and functional responses to diflufenican exposure have not been resolved. Moreover, how diflufenican alone modulates bacterial community assembly and functional potential across the soil profile remains insufficiently understood. Consequently, depth-resolved investigations grounded in the genetic horizon framework are essential for accurately assessing the ecological implications of herbicide residues. However, information regarding how diflufenican perturbation affects bacterial communities across different soil depths remains limited, and the short-term community- and functional-level responses along the vertical profile following diflufenican exposure remain largely unexplored.

Here, we investigated depth-resolved bacterial community and functional responses to diflufenican perturbation in agricultural soils from two wheat-producing regions in Inner Mongolia. By integrating 16S rRNA gene amplicon sequencing with FAPROTAX and PICRUSt2 functional prediction [29,30,31,32,33], we tested three specific hypotheses: (i) diflufenican exposure will drive divergent bacterial community responses across the soil depth gradient, with deeper soil horizonsexhibiting greater community dissimilarity from the control compared to topsoil; (ii) taxa associated with xenobiotic tolerance and anaerobic metabolism will exhibit differential enrichment across the soil depth gradient following diflufenican exposure, reflecting depth-specific community adaptation strategies to chemical stress; (iii) denitrification and fermentation pathways will be inferred as enriched in diflufenican-treated deep soils (30–90 cm) relative to topsoil, because diflufenican-induced oxygen depletion and organic carbon limitation will select for facultative anaerobic metabolisms. We anticipated that diflufenican perturbation would reshape bacterial community structure and alter inferred functional potential in a depth-dependent manner, highlighting the importance of vertical soil depth in assessing herbicide impacts on agricultural soil microbiomes.

## 2. Materials and Methods

### 2.1. Soil Sampling and Experimental Design

Soil samples were collected from six agricultural fields located in Ulanqab (40°59′ N, 113°07′ E) and Tongliao (43°39′ N, 122°14′ E), Inner Mongolia, China, in April 2022 (Figure S1). The regional climate is characterized as a semi-arid temperate continental monsoon climate, with cold dry winters, warm wet summers, mean annual temperature ranging from approximately 0 to 18 °C, annual precipitation of 150–450 mm, and a frost-free period of approximately 95–145 d [34]. The annual evaporation in Ulanqab City is 1740–3000 mm, and the annual evaporation in Horqin District of Tongliao City is 1068–2200 mm. The soils in Ulanqab are chestnut soils (Calcic Kastanozems in WRB; Typic Haplocalcids in USDA Soil Taxonomy), while those in Tongliao are black soils (Haplic Chernozems in WRB; Typic Haplocryolls in USDA Soil Taxonomy) [35,36]. Both sites had been under continuous rain-fed wheat monoculture for at least ten years prior to sampling, with conventional moldboard plowing to 18–22 cm and occasional subsoiling to 25–30 cm, according to local field management records.

At each sampling location, a 1.2 m tubular auger was employed to extract soil core samples from representative plots following a grid-based sampling pattern. The auger was inserted into the soil to a depth of approximately one meter and subsequently retrieved. Soil samples corresponding to specific depth intervals were collected based on the graduated markings on the instrument. Soil samples were collected from three depth intervals: 0–30 cm (topsoil), 31–60 cm (subsoil), and 61–90 cm (deep soil), resulting in a total of 18 independent composite samples (6 sites × 3 depths). For each depth interval at each site, multiple subsamples were randomly collected and thoroughly homogenized to constitute one composite sample. Visible roots, stones, and plant residues were removed manually. The composite soils were then sieved through a 2 mm mesh and stored at 4 °C; sterile distilled water was periodically sprayed to maintain soil moisture when necessary. Prior to the microcosm incubation, each composite sample was divided into two portions: one serving as the control treatment (CK, without diflufenican addition) and the other as the diflufenican-amended treatment (DI). Each experimental group had 6 replicates, three control groups and three treatment groups, generating 36 experimental units in total. Table S1 presents the fundamental physicochemical properties of soil samples collected from six sampling sites at three distinct depths.

### 2.2. Preliminary Enrichment Assay

Before the microcosm experiment, a preliminary enrichment assay was conducted to verify that the selected diflufenican concentration would permit detectable microbial growth, following the general rationale of pesticide-enrichment assays used to evaluate microbial growth under herbicide exposure [37,38]. Briefly, 5 g of each soil sample (*n* = 18) was suspended in 0.85% (*w*/*v*) sterile physiological saline and shaken at 180 rpm for 30 min. The supernatant (1 mL) was collected after static incubation and inoculated into mineral salt medium supplemented with diflufenican at concentrations of 50, 100, and 150 mg·L^−1^. The cultures were then incubated in the dark at 30 °C with shaking at 180 rpm for 6 days. Microbial growth was monitored every 24 h by measuring optical density at 600 nm (OD_600_), and the relative OD increase was calculated on day 6 as follows:

Growth rate (%) = (OD_600,day6_ − OD_600,day0_)/OD_600,day0_ × 100

Positive OD increases across all six sampling sites indicated that 50 mg·L^−1^ diflufenican did not completely inhibit detectable microbial growth of the indigenous soil microbiota (Figure S2), supporting the selection of this concentration for subsequent microcosm experiments. This assay was used solely as a preliminary observation of microbial viability under diflufenican exposure and was not intended to determine a toxicity threshold or to confirm diflufenican degradation capacity.

### 2.3. Soil Microcosm Incubation

A short-term soil microcosm experiment was conducted to investigate bacterial community responses to diflufenican exposure across different soil depths. For each composite soil sample, 50 g of fresh soil was transferred into sterile incubation containers. The diflufenican-treated group (DI) received an aqueous diflufenican solution at a concentration of 50 mg L^−1^, whereas the control group (CK) received the same volume of sterile water without diflufenican. All microcosms were incubated in the dark at 30 °C for six days. At the end of incubation, soil samples were collected immediately. For each microcosm, 1 g of soil was subsampled, snap-frozen in liquid nitrogen, and stored at −80 °C for subsequent DNA extraction and 16S rRNA gene amplicon sequencing.

### 2.4. DNA Extraction and 16S rRNA Gene Amplicon Sequencing

Total genomic DNA was extracted from 0.5 g of each frozen soil sample using the PowerSoil DNA Isolation Kit (MO BIO Laboratories, Carlsbad, CA, USA) according to the manufacturer’s instructions. DNA concentration and purity were assessed by spectrophotometry and 1% (*w*/*v*) agarose gel electrophoresis. Qualified DNA samples were stored at −20 °C until further analysis. The V3–V4 hypervariable region of the bacterial 16S rRNA gene was amplified using primers 338F (5′-ACTCCTACGGGAGGCAGCAG-3′) and 806R (5′-GGACTACHVGGGTWTCTAAT-3′), which are commonly used for bacterial community profiling [29,39]. Detailed PCR amplification and library construction were performed following standard Illumina amplicon sequencing workflows [29,30]. The resulting amplicon libraries were sequenced on the Illumina NovaSeq platform (Allwegene Co., Ltd., Harbin, China) using a paired-end sequencing strategy.

### 2.5. Bioinformatics Analysis

Raw sequencing reads were quality-filtered, merged, and denoised. Chimeric sequences were removed, and operational taxonomic units (OTUs) were clustered at 97% sequence similarity using QIIME (Version 1.8.0 http://qiime.org/ (accessed on 20 May 2022). Representative sequences were taxonomically assigned against the SILVA database. All samples were rarefied to an even sequencing depth prior to downstream analysis.

Alpha diversity indices, including observed OTUs (species richness), Shannon diversity, and Simpson diversity, were calculated using QIIME. Beta diversity was assessed based on Bray–Curtis dissimilarity matrices derived from taxonomic relative abundances. Principal coordinate analysis (PCoA) was performed to visualize differences in bacterial community composition among treatments. Analysis of similarities (ANOSIM) was conducted using the vegan package (v2.5-7) in R (v3.6.3) to test group separation significance [40,41]. Differential OTUs were visualized using Manhattan plots, with phylum-level taxonomy on the *x*-axis and OTU abundance on the *y*-axis [42]. Linear discriminant analysis effect size (LEfSe) was applied to identify key discriminant taxa across soil depths and diflufenican treatments.

### 2.6. Co-Occurrence Network Analysis

Co-occurrence networks were constructed to explore potential association patterns among bacterial taxa. Low-abundance and low-frequency taxa were removed before network construction. Pairwise correlations among bacterial taxa were calculated using Spearman. Edges were retained for strong and significant correlations (Spearman |ρ| > 0.9, *p* < 0.05). Network graphs were constructed and visualized using Gephi (v0.9-2) [43]. Network topological properties, including weighted robustness, stability, positive and negative cohesion, and vulnerability, were evaluated using the ggClusterNet package (v0.1-0) in R [44].

### 2.7. Functional Prediction

Bacterial functional potential was inferred from 16S rRNA gene amplicon data using two complementary approaches. Phylogenetic Investigation of Communities by Reconstruction of Unobserved States 2 (PICRUSt2) was used to predict Kyoto Encyclopedia of Genes and Genomes orthologs and compare functional potentials between CK and DI treatments [31]. Functional Annotation of Prokaryotic Taxa (FAPROTAX) was further used to infer putative ecological functions of bacterial taxa, particularly those related to carbon and nitrogen cycling [32,33]. Predicted functional profiles were subsequently analyzed to identify treatment- and depth-dependent shifts.

### 2.8. Statistical Analysis

All statistical analyses were performed in R version 3.6.3 unless otherwise specified [45]. Permutational multivariate analysis of variance (PERMANOVA) was conducted with 9999 permutations using the adonis2 function in the vegan package version 2.5-7 to assess the significance of community compositional differences among diflufenican treatments and soil depths [46,47]. Pairwise comparisons were performed where applicable. The significance threshold was set at *p* < 0.05 for all tests. Where multiple comparisons were performed, *p* values were adjusted using the Benjamini–Hochberg false discovery rate method [48].

## 3. Results

### 3.1. Diflufenican Reduced Bacterial Richness in a Depth-Dependent Manner

After quality filtering, a total of 1,876,089 high-quality reads were obtained from the 36 soil samples, clustering into 5360 bacterial operational taxonomic units (OTUs) at 97% sequence similarity. Rarefaction curves approached saturation for all samples, indicating that the sequencing depth was sufficient to capture the majority of community diversity (Figure S3).

**Figure 1:**
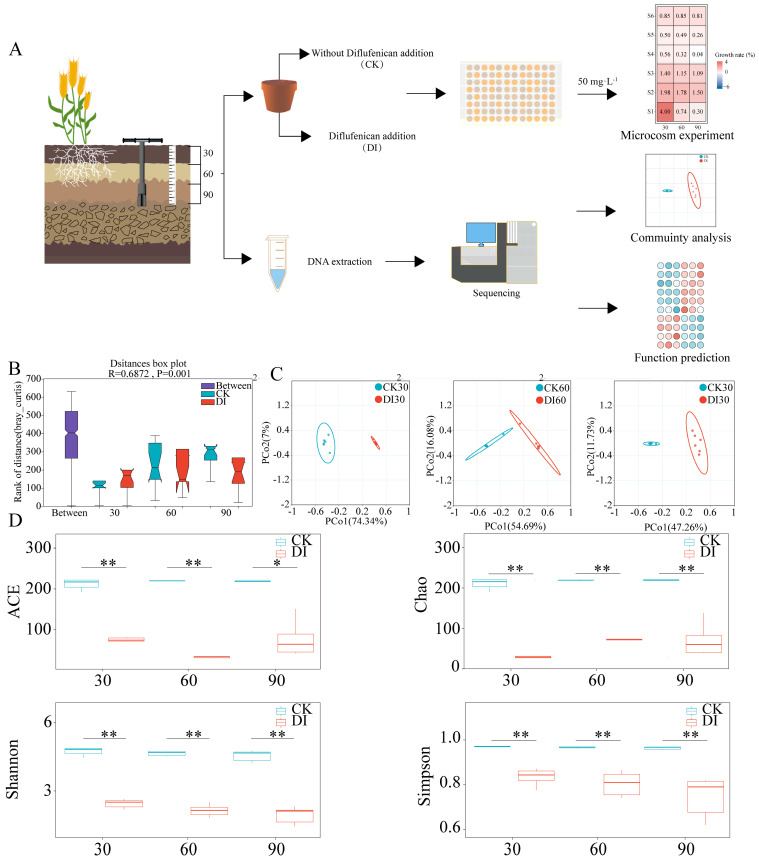
Effects of diflufenican exposure on bacterial community structure and alpha diversity across soil depths. (**A**) Sampling scheme and experimental design. Soil samples were collected from three depth intervals (0–30 cm, 31–60 cm, and 61–90 cm) and incubated under control (CK) or diflufenican (DI) conditions for six days. (**B**) Analysis of similarities (ANOSIM) based on Bray–Curtis dissimilarity. (**C**) Principal coordinate analysis (PCoA) of bacterial communities based on Bray–Curtis distances. Ellipses represent 95% confidence intervals. (**D**) Alpha diversity indices (ACE, Chao1, Shannon, and Simpson) comparing CK and DI treatments at each soil depth. Asterisks indicate significant differences between CK and DI at the same depth: * *p* < 0.05, ** *p* < 0.01. Different lowercase letters indicate significant differences among depths within the same treatment (Tukey’s honestly significant difference test). Blue and red colors indicate the control treatment (CK, without diflufenican addition) and the diflufenican-amended treatment (DI), respectively.

Diflufenican exposure significantly altered bacterial community structure across the soil profile. Analysis of similarities (ANOSIM) revealed robust separation between control (CK) and diflufenican-treated (DI) groups (R = 0.69, *p* = 0.001; Figure 1B). Principal coordinate analysis (PCoA) based on Bray–Curtis dissimilarity further demonstrated clear treatment-associated partitioning along the primary axis (Figure 1C). Notably, CK samples exhibited a gradual depth-dependent clustering pattern, whereas DI samples displayed increasing divergence with soil depth, indicating that diflufenican disrupted the natural vertical stratification of bacterial communities.

Alpha diversity analyses revealed that diflufenican reduced bacterial richness and diversity across all depths, but the pattern of reduction was depth-dependent (Figure 1D). ACE and Chao1 indices indicated substantial species loss following treatment. The 60 cm layer exhibited the most pronounced reduction in species number, whereas the 30 cm layer maintained the lowest absolute richness. At 90 cm, species number approached that of 30 cm (ACE) or 60 cm (Chao1), indicating convergent impoverishment in deeper horizons under herbicide stress. Shannon and Simpson indices also declined, reflecting not only reduced diversity but also altered community evenness, suggesting that diflufenican selectively eliminated sensitive taxa and allowed stress-tolerant populations to dominate.

### 3.2. Diflufenican Altered Bacterial Community Composition

**Figure 2:**
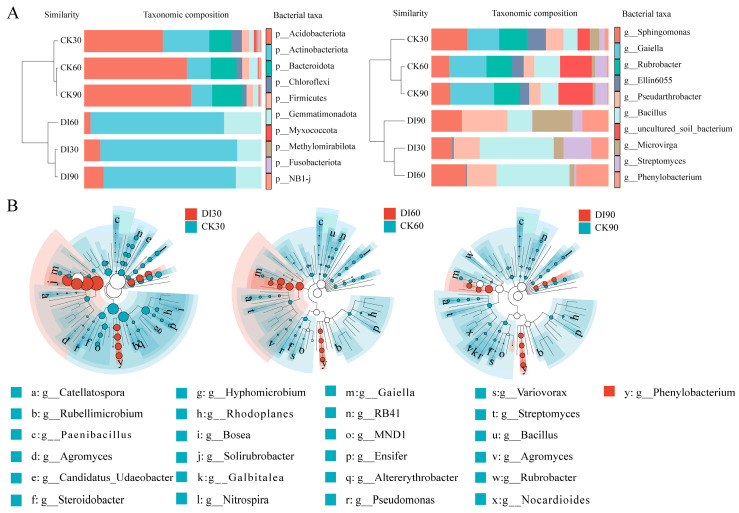
Taxonomic shifts in bacterial communities following diflufenican exposure across soil depths. (**A**) UPGMA clustering dendrogram based on Bray–Curtis distances showing separation between CK and DI communities. (**B**) Cladogram generated by linear discriminant analysis effect size (LEfSe) showing the phylogenetic distribution of bacterial lineages discriminating CK (blue) and DI (red). Taxa with linear discriminant analysis (LDA) scores > 2.5 are indicated.

Taxonomic profiling revealed pronounced divergence between control and diflufenican-treated soils across the bacterial phylogeny. At the phylum level, Actinobacteriota and Gemmatimonadota emerged as the primary responders, increasing in relative abundance under treatment, whereas Acidobacteriota and most other phyla declined (Figure 2A, Figures S4 and S5). Vertical heterogeneity modulated these responses: Actinobacteriota enrichment was strongest at 30 cm and 90 cm, while Gemmatimonadota peaked at 60 cm, followed by 90 cm and 30 cm.

Finer-scale analysis at the genus level further resolved these depth-stratified treatment effects. Diflufenican consistently enriched putatively stress-tolerant taxa, including *Sphingomonas*, *Pseudarthrobacter*, *Bacillus*, *Microvirga*, *and Phenylobacterium*, while depleting *Gaielia*, *Rubrobacter*, *Ellin6055*, and *Streptomyces* (Figure 2A). UPGMA clustering based on Bray–Curtis distances and Circos plots illustrated clear separation between CK and DI communities across the soil profile.

### 3.3. Diflufenican-Associated Taxa Differed Among Soil Depths

The vertical distribution of responsive taxa exhibited distinct depth-specific signatures (Figure 2A). *Rubrobacter* showed a U-shaped abundance pattern under diflufenican exposure, with relatively higher abundance retained at 30 cm and 90 cm and lowest abundance at 60 cm, despite overall reduction across all depths. In contrast, *Sphingobacterium* increased under diflufenican exposure, with enrichment peaking at 60 cm, followed by 90 cm and 30 cm. *Gaielia* and *Ellin6055* both showed highest relative abundance at 60 cm under diflufenican exposure, with lower representation at 30 cm and 90 cm, suggesting preferential retention in the subsoil layer despite overall depletion. *Pseudarthrobacter* and *Bacillus* were most enriched at 30 cm and decreased with depth, whereas *Microvirga* and *Streptomyces* maintained higher abundance at 30 cm and 60 cm but dropped sharply at 90 cm. *Phenylobacterium* exhibited strongest enrichment at 60 cm, with lower abundance in surface and deep soils.

To identify robust biomarkers transcending depth effects, linear discriminant analysis effect size (LEfSe) was performed with a logarithmic LDA score threshold of 2.5. Only *Phenylobacterium* achieved statistical significance as a discriminant taxon distinguishing diflufenican-treated soils from controls (Figure 2B). This biomarker signature remained consistent across all three depths, indicating that *Phenylobacterium* represents a depth-independent indicator of diflufenican perturbation. Collectively, these findings demonstrate that diflufenican-associated taxa are not uniformly distributed along the soil profile but exhibit depth-stratified responses, reflecting the interplay between herbicide stress and vertical soil heterogeneity.

### 3.4. Co-Occurrence Patterns Changed After Diflufenican Exposure

**Figure 3:**
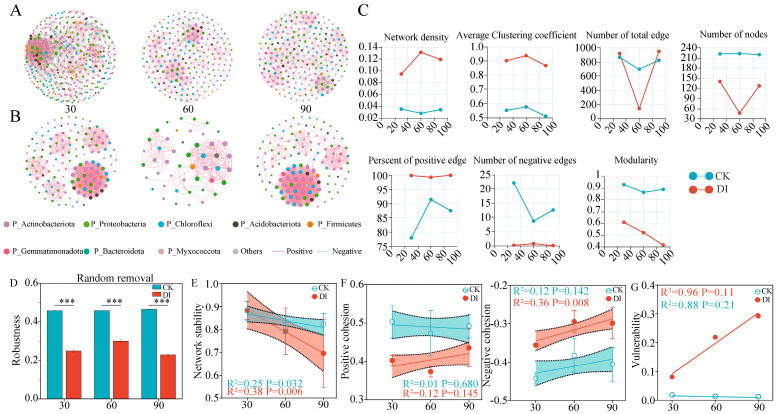
Co-occurrence network topology and stability of bacterial communities under diflufenican exposure. (**A**,**B**) Bacterial co-occurrence networks in CK (**A**) and DI (**B**) soils. Nodes represent bacterial genera; edges represent significant Spearman correlations (Spearman |ρ| > 0.9, *p* < 0.05). Positive and negative correlations are shown in red and green, respectively. (**C**) Topological properties including network density, average clustering coefficient, percentage of positive edges, number of negative edges, number of total edges, number of nodes, and modularity. Values are provided in Table S1. (**D**) Network robustness assessed as the proportion of taxa remaining after random removal of 50% of nodes from each empirical network (*** *p* < 0.001). (**E**) Network stability across soil depths. Linear regression parameters (slope b, adjusted R^2^, and *p* values) are shown. (**F**) Positive and negative cohesion of bacterial communities. (**G**) Network vulnerability measured by maximum node vulnerability. Linear regression statistics (adjusted R^2^ and *p* values) are shown. Blue and red colors indicate the control treatment (CK, without diflufenican addition) and the diflufenican-amended treatment (DI), respectively.

Beyond taxonomic shifts, diflufenican exposure profoundly restructured bacterial inter-species association networks. Co-occurrence network analysis showed that treatment networks exhibited fewer nodes and edges than control networks across all depths; concomitantly, the remaining nodes formed highly clustered local modules, yielding higher network density but lower average degree (Figure 3C; Table S2). In CK soils, the 60 cm layer harbored the strongest inter-taxa associations, whereas under DI, 30 cm and 90 cm networks exceeded 60 cm in connectivity, indicating that the subsoil community was disproportionately destabilized. Concomitantly, the average clustering coefficient at 60 cm increased under treatment, reflecting localized aggregation of surviving taxa. The proportion of positive correlations increased after diflufenican exposure, yet positive cohesion remained unchanged (CK: R^2^ = 0.12, *p* = 0.680; DI: R^2^ = 0.12, *p* = 0.145), whereas negative cohesion—indicative of competitive or antagonistic interactions—weakened significantly (R^2^ = 0.36, *p* = 0.008; Figure 3F). Network modularity also declined. These changes suggest a shift from balanced competition–cooperation toward depauperate, loose coexistence rather than enhanced synergism.

To quantify the consequences of these topological alterations for ecosystem resilience, robustness was assessed by simulating random taxon removal. CK networks exhibited comparable resilience across depths, whereas DI networks displayed significantly reduced robustness (Figure 3D). Network stability decreased with soil depth under diflufenican, with a markedly steeper stability slope in DI than CK (Figure 3E), indicating that deeper communities were more vulnerable to perturbation. Network vulnerability remained near zero in CK soils, reflecting high redundancy, but increased sharply under diflufenican (R^2^ = 0.96; Figure 3G), suggesting that the loss of dominant taxa triggered network collapse. Together, these results demonstrate that diflufenican-induced species loss reduced competitive pressure, increased the relative proportion of positive associations, and forced surviving bacteria to persist via niche avoidance rather than active cooperation.

### 3.5. Predicted Functional Profiles Shifted Under Diflufenican Exposure

**Figure 4:**
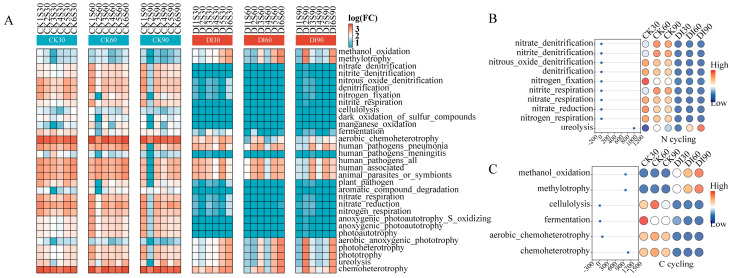
FAPROTAX-predicted functional shifts in bacterial communities following diflufenican exposure. (**A**) FAPROTAX-predicted functional profiles showing relative abundances of biogeochemical cycling-related functions across treatments and depths. (**B**) Nitrogen cycling functions inferred by FAPROTAX. Bubble size represents relative abundance; color intensity indicates the magnitude of enrichment. (**C**) Carbon metabolism and energy acquisition functions predicted by FAPROTAX.

To assess ecosystem-level functional consequences, FAPROTAX was used to infer biogeochemical cycling capacities from taxonomic composition. Diflufenican induced severe functional imbalance across the soil profile (Figure 4A). CK soils maintained functionally diverse communities that gradually simplified with depth. In contrast, DI soils showed aberrant enrichment of pathogen-related functions and sulfur/manganese oxidation, alongside systematic suppression of nitrogen fixation, phototrophy, and cellulose decomposition in deeper layers, indicating a loss of metabolic flexibility under herbicide stress. Nitrogen cycling functions were substantially disrupted. Nitrogen fixation was inhibited at all depths, whereas denitrification and nitrite respiration were significantly enhanced at 60–90 cm (Figure 4B). Carbon cycling profiles indicated a fundamental metabolic shift: cellulose decomposition capacity weakened progressively with depth, while fermentation and methanol oxidation became relatively enriched in deep soils (Figure 4C).

**Figure 5:**
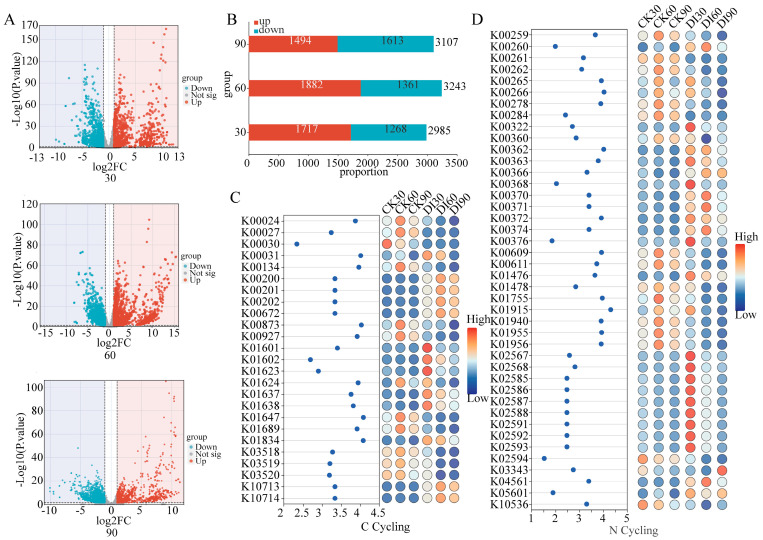
PICRUSt2-predicted functional gene abundance in bacterial communities following diflufenican exposure. (**A**) Differentially abundant PICRUSt2 functions prediction between CK and DI treatments. (**B**) PICRUSt2-predicted differentially abundant KEGG orthologs (KOs) between CK and DI treatments at each depth. Numbers indicate up- and down-regulated KOs. (**C**) Heatmap of PICRUSt2-predicted carbon metabolism enzyme abundances (KEGG orthologs). (**D**) Heatmap of PICRUSt2-predicted nitrogen metabolism enzyme abundances. The KEGG Orthology (KO) annotation information is shown in Table S2.

These FAPROTAX-inferred shifts were corroborated by PICRUSt2-based prediction of KEGG ortholog abundances. PCoA of predicted functional β-diversity demonstrated clear separation between CK and DI communities across depths (Figure S6). Volcano plots revealed depth-dependent differences in the number of differentially predicted genes: 60 cm exhibited the largest response (3243 KOs; 1882 up, 1361 down), followed by 90 cm (3107 KOs; 1494 up, 1613 down) and 30 cm (2985 KOs; 1717 up, 1268 down) (Figure 5A,B).

At the metabolic pathway level, carbon metabolism enzymes exhibited distinct vertical stratification under diflufenican exposure (Figure 5C). Calvin cycle enzymes—ribulose-1,5-bisphosphate carboxylase/oxygenase large subunit (*rbcL*/K01601) and small subunit (*rbcS*/K01602)—were elevated at 30 cm but low at 60 cm and 90 cm, suggesting enhanced autotrophic carbon fixation potential in surface soils. Isocitrate dehydrogenase (IDH1/K00031), indicative of tricarboxylic acid cycle activity, increased at 30 cm and 60 cm. Glycolytic and glyoxylate cycle enzymes, including fructose-bisphosphate aldolase (ALDO/K01623, FBA/K01624), malate synthase (*aceA*/K01637), and isocitrate lyase (aceB/K01638), peaked at 30 cm, pointing to intensified organic carbon catabolism in surface soils. Conversely, reductive acetyl-CoA pathway enzymes—formyltetrahydrofolate synthetase (*fwdA*/K00200) and associated components (*fwdB*/K00201, *fwdC*/K00202, *ftr*/K00672, *fae*/K10713, *mtdB*/K10714)—were most abundant at 60 cm, implying active hydrogenotrophic methanogenesis or acetogenic carbon fixation in subsoil horizons.

Nitrogen metabolism enzymes exhibited depth-differentiated responses under diflufenican stress (Figure 5D). Dissimilatory nitrogen loss pathways were prominently enriched in upper and middle soil profiles. Respiratory nitrate reductases (*narG*/K00370, *narH*/K00371) were elevated at 30 cm and 60 cm, as were their membrane anchor subunit (*narI*/K00374) across all three depths. Nitrous oxide reductase (*nosZ*/K00376), catalyzing the terminal denitrification step, showed enrichment specifically at 30 cm. DNRA-associated nitrite reductases (*nirB*/K00362, *nirD*/K00363) also increased at 30 cm and 60 cm, indicating potential for nitrate reduction to ammonium in upper-middle layers. Nitrogen assimilation pathways displayed distinct depth patterns. Assimilatory nitrate reductase (*nasA*/K00372) and urease (*ureC*/K01476) were enriched across all depths (30–90 cm), suggesting sustained capacity for nitrate and organic nitrogen utilization throughout the soil profile. Ammonia incorporation enzymes—glutamine synthetase (*glnA*/K01915) and glutamate synthases (*gltB*/K00265, *gltD*/K00266)-were elevated at 30 cm, pointing to localized glutamine/glutamate synthesis capacity in the upper layer. Ferredoxin-nitrite reductase (*nirA*/K00322) was also enriched at 30 cm. Nitrogen fixation components showed a restricted distribution: nitrogenase iron protein (*nifH*/K02588) and core structural genes (*nifD*/K02585, *nifK*/K02586, *nifE*/K02587, *nifN*/K02591, *nifX*/K02592, *nifW*/K02593), along with regulatory proteins (*nifA*/K02567, *nifL*/K02568), were enriched specifically at 30 cm, while accessory proteins *nifU* (K04561) extended to 60 cm and *nifS* (K05601) to all depths. Hydroxylamine oxidoreductase (*hao*/K00260), involved in nitrification, was enriched at 30 cm and 60 cm.

## 4. Discussion

### 4.1. Diflufenican Exposure Reshaped Bacterial Communities in a Depth-Dependent Manner

Soil bacterial communities exhibit depth-dependent responses to external environmental perturbations, and changes in deeper soil communities cannot be inferred from those in surface layers [19]. Column experiments have revealed the redistribution of glyphosate from the surface to deeper soil strata, with its concentration progressively diminishing as it penetrates deeper. Consequently, under natural application conditions, the microbial degradation of glyphosate is primarily associated with the upper soil horizons [49]. Although herbicide-induced microbial responses in soil systems have been increasingly studied, research explicitly examining vertical microbial patterns under herbicide disturbance remains limited. For instance, comparable evidence regarding diflufenican is still scarce. Existing studies on diflufenican predominantly focus on herbicide dissipation, surface soil microbial activity, or degrading strains [22,38], yet lack a comprehensive assessment of bacterial community composition, co-occurrence patterns, and predictive functional potential across the entire agricultural soil profile. However, recent evidence suggests that even surface-adsorbed herbicides can exert legacy effects through vertical transport of transformation products or prolonged bioavailability in adjacent surface layers [8]. Investigating the response of deeper soil bacterial communities to diflufenican is crucial for understanding their response and degradation potential. Therefore, in this study, we employed microcosm experiments to examine the effects of uniform concentrations of diflufenican application on bacterial communities across different soil depths.

Our findings indicate that, under standardized diflufenican dosages, communities from intermediate soil profiles exhibit disproportionate vulnerability relative to those from upper profiles, manifested as lower taxonomic diversity and diminished functional insurance. This differential sensitivity is intrinsic to the community characteristics at each depth, rather than a consequence of differential herbicide exposure. Deeper microbial communities are typically characterized by resource scarcity, lower functional redundancy, and slower growth rates [14,16], which aligns with the ecological principle that long-term resource-limited communities possess reduced resilience to acute chemical stress [50]. This is consistent with the broader ecological tenet that chronically resource-constrained subsoil communities exhibit lower resistance to acute chemical perturbations.

### 4.2. Enriched Taxa May Represent Candidate Diflufenican-Associated Bacteria

The phylum-level restructuring observed in this study, characterized by enrichment of Actinobacteriota and Gemmatimonadota together with Acidobacteriota depletion, is broadly consistent with the selective responses of soil bacterial communities to herbicide disturbance. For example, fomesafen application was reported to increase the relative abundance of Proteobacteria and Actinobacteria while decreasing Verrucomicrobia in soybean soils [51]. Butachlor exposure was also shown to alter soil bacterial community composition, with decreased Acidobacteriota and increased Proteobacteria in butachlor-contaminated soil, although the direction of response for other phyla depended on remediation stage and bacterial inoculation [52]. The depth-specific peak of Gemmatimonadota at 60 cm suggests that subsoil communities may recruit distinct stress-adapted lineages under diflufenican exposure, potentially reflecting the hydrophobic and slowly degrading nature of this herbicide.

At finer taxonomic resolution, the enrichment of *Sphingomonas*, *Pseudarthrobacter*, *Bacillus*, *Microvirga*, and *Phenylobacterium* across soil depths suggests co-enrichment of taxa potentially tolerant to diflufenican-amended conditions. Members of *Sphingomonas* and *Pseudarthrobacter* have been reported to degrade aromatic compounds, including polycyclic aromatic hydrocarbons [53,54], while *Bacillus* strains have been associated with the degradation of herbicides such as prometryn and acetochlor [55]. Notably, LEfSe identified *Phenylobacterium* as a cross-depth biomarker. This genus is of particular interest because Phenylobacterium immobile was originally described as capable of utilizing xenobiotic compounds such as chloridazon as sole carbon sources [56], and pyrazon oxygenase from this species catalyzes dioxygenase-mediated hydroxylation [57]. We therefore propose *Phenylobacterium* as a hypothesis-generating biomarker for subsequent culture-dependent and genome-resolved studies. Future work should combine isolation, residue dissipation assays, and metabolite identification to determine whether this genus directly contributes to diflufenican transformation.

### 4.3. Co-Occurrence Network Destabilization Reflects Altered Ecological Interactions

While taxonomic shifts indicate which bacterial groups responded to diflufenican exposure, co-occurrence network analysis provides an exploratory view of potential association patterns among soil microbial taxa [58] and how these patterns may change under herbicide disturbance. In this study, diflufenican exposure altered network topological properties, including node number, edge number, modularity, cohesion, and vulnerability, suggesting that bacterial association patterns were restructured after diflufenican exposure. Recent work has shown that pesticide residues can reduce bacterial diversity while enhancing network stability through motif-level restructuring under chronic greenhouse contamination conditions [59]. In contrast, our results suggest that diflufenican exposure was associated with reduced network complexity and increased vulnerability, particularly in deeper soil horizons. This discrepancy may reflect differences in exposure context, pesticide composition, soil nutrient status, and baseline microbial community structure. The greenhouse soils examined by them represented a chronically contaminated and nutrient-enriched system, whereas the present microcosm experiment involved short-term exposure of depth-specific agricultural soils [59]. Therefore, network responses to pesticide stress may depend strongly on soil context and disturbance history.

The decrease in negative cohesion without a corresponding increase in positive cohesion may indicate a weakening of the balance between negative and positive association patterns. Ecological theory suggests that competitive or antagonistic interactions can contribute to microbial community stability by limiting positive feedbacks and preventing the dominance of cooperative subnetworks [60]. However, because correlation-based networks do not directly identify competition or cooperation, the observed reduction in negative associations should be interpreted cautiously. The stronger network response in deeper soils may be related to the lower diversity, reduced resource availability, and distinct physiological status of subsoil microbial communities. This interpretation is consistent with the broader view that soil microbial resistance and resilience are shaped by soil physicochemical structure, microbial community composition, and disturbance history [50]. Collectively, these findings suggest that herbicide impact assessments should not rely solely on diversity metrics, but should also consider potential changes in microbial association architecture, particularly in deeper soil horizons.

### 4.4. Predicted Functional Shifts Indicate Potential Changes in Nutrient Cycling

The predicted shift in nitrogen cycling functions suggests that diflufenican exposure may alter the functional potential of bacterial communities involved in soil N transformations, a phenomenon consistent with herbicide-induced changes in microbial N cycling reported in other systems. For example, mesotrione was shown to inhibit functional genes involved in nitrification, denitrification, and assimilatory nitrate reduction while promoting genes related to nitrogen fixation in a greenhouse soil experiment [61]. Butachlor application was also reported to increase N_2_O emissions in dry-seeded rice fields, potentially through changes in mineral nitrogen availability and the abundance of ammonia-oxidizing and denitrifying bacteria [62]. Our finding that diflufenican enhances denitrification at 60–90 cm while suppressing fixation at all depths suggests the herbicide-class-specific effect: pyridinecarboxamide herbicides may create localized anaerobic microsites that favor dissimilatory over assimilatory N-pathways. This is further supported by the concurrent enrichment of fermentation and methanol oxidation functions, indicating a metabolic shift from aerobic chemoorganotrophy toward anaerobic/facultative methylotrophy.

The predicted changes in carbon metabolism further suggest that bacterial communities from different soil depths may adopt distinct functional strategies under diflufenican exposure. PICRUSt2 indicated enrichment of predicted Calvin cycle- and glycolysis-related enzymes at 30 cm, whereas predicted functions associated with the reductive acetyl-CoA pathway were more evident at 60 cm. These depth-specific patterns may reflect differences in resource availability and baseline microbial composition between surface-adjacent and subsoil horizons. Surface communities may intensify autotrophic and organotrophic catabolism to exploit labile carbon from decaying sensitive taxa, while subsoil communities appear to rely on hydrogenotrophic or acetogenic fixation, possibly reflecting energy limitation in deeper horizons. Notably, the suppression of cellulose decomposition across depths contrasts with the maintenance of urease activity, suggesting that diflufenican selectively inhibits recalcitrant carbon degradation while sparing mineral nitrogen mobilization—a pattern that could accelerate soil carbon depletion over repeated applications.

## 5. Conclusions

This study demonstrates that diflufenican exposure induces depth-dependent restructuring of soil bacterial communities and alters inferred functional potential across the 0–90 cm agricultural soil profile. Under standardized incubation conditions, subsoil communities (60–90 cm) exhibited disproportionate vulnerability, with the 60 cm layer showing the most pronounced species loss. Taxonomic analysis revealed consistent enrichment of stress-tolerant taxa—notably *Phenylobacterium*, which was consistently responsive across all soil horizons—suggesting its potential as a candidate biomarker for diflufenican perturbation. Co-occurrence networks shifted from structured, highly connected assemblages toward depauperate, loosely connected coexistence, characterized by weakened competitive interactions without enhanced cooperative cohesion, with disproportionate destabilization in deeper horizons where functional redundancy is inherently lower. Inferred functional profiles indicated suppression of nitrogen fixation and phototrophy alongside relative enhancement of denitrification and fermentation, with depth-specific divergence in carbon acquisition strategies—Calvin cycle and glycolytic enzymes prevailing at 30 cm, and reductive acetyl-CoA pathway enzymes at 60 cm—suggesting a metabolic redirection toward anaerobic and methylotrophic strategies under herbicide stress.

## Data Availability

The original contributions presented in this study are included in the article/Supplementary Material. Further inquiries can be directed to the corresponding authors.

## Supplementary Materials

The following supporting information can be downloaded at: https://www.mdpi.com/article/10.3390/microorganisms14071531/s1, Figure S1: Detailed geographic coordinates of sampling sites in Ulanqab City, western Inner Mongolia, China. Sites marked in red were used for soil sampling in this study; Figure S2: Microbial growth rates at different soil depths and sampling sites under different concentrations of diflufenican exposure; Figure S3: Species dilution curve; Figure S4: Community structure in treatment and control group; Figure S5: Circos plot at the level of the grouping genus classification; Figure S6: PcoA analysis based on PICRUSt2 functional prediction; Table S1: Soil physical and chemical properties at different depths of the sampling point. Table S2: Characterization of microbial network interactions before and after treatment; Table S3: PICRUSt2 carbon and nitrogen metabolism function prediction KEGG Orthology (KO) annotation information.

## Abbreviations

The following abbreviations are used in this manuscript:

CK	Without diflufenican addition
DI	Diflufenican-amended treatment
