# Supplementary material for: Diflufenican Perturbation Reshapes Bacterial Community Structure and Alters Functional Potential Across Agricultural Soil Depths
Source: Microorganisms. 2026 Jul 13;14(7):1531. doi: 10.3390/microorganisms14071531 (PMC13413908; doi:10.3390/microorganisms14071531)
Supplement: Supplementary file 1 [file microorganisms-14-01531-s001.zip › microorganisms-4383906-supplementary.pdf]

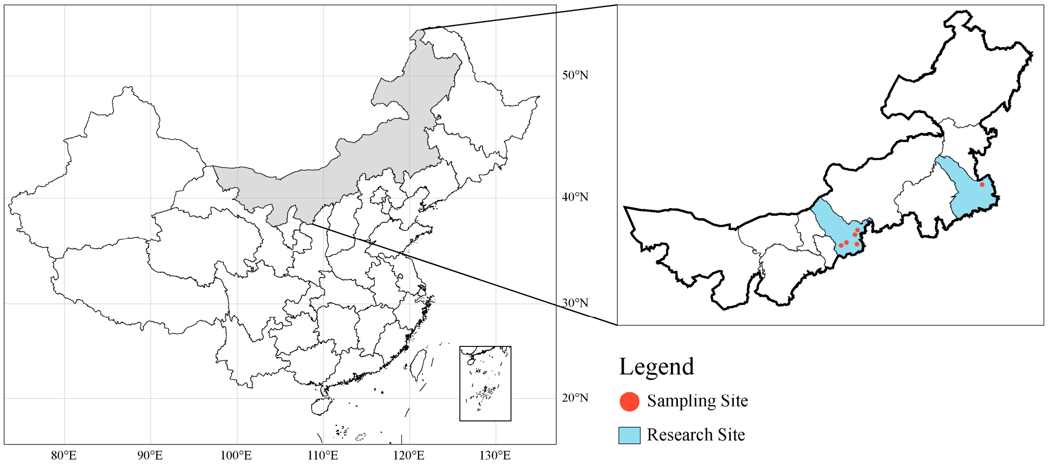

Figure S1. Detailed geographic coordinates of sampling sites in Ulanqab City, western Inner Mongolia, China. Sites marked in red were used for soil sampling in this study.

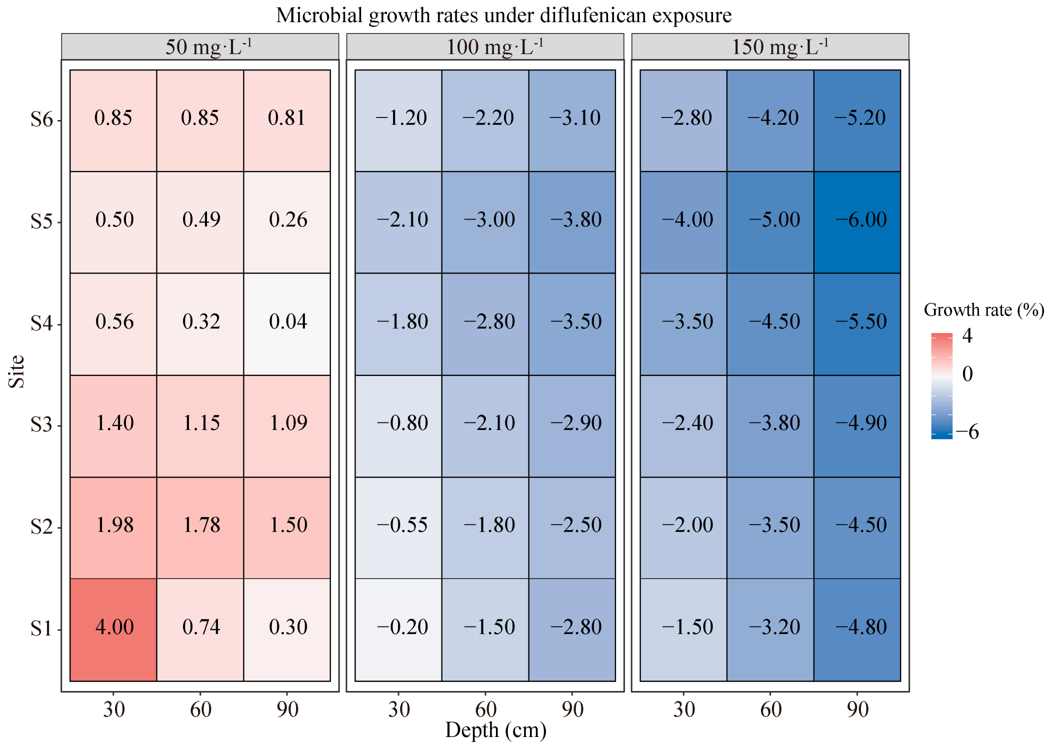

Figure S2. Microbial growth rates at different soil depths and sampling sites under different concentrations of diflufenican exposure.

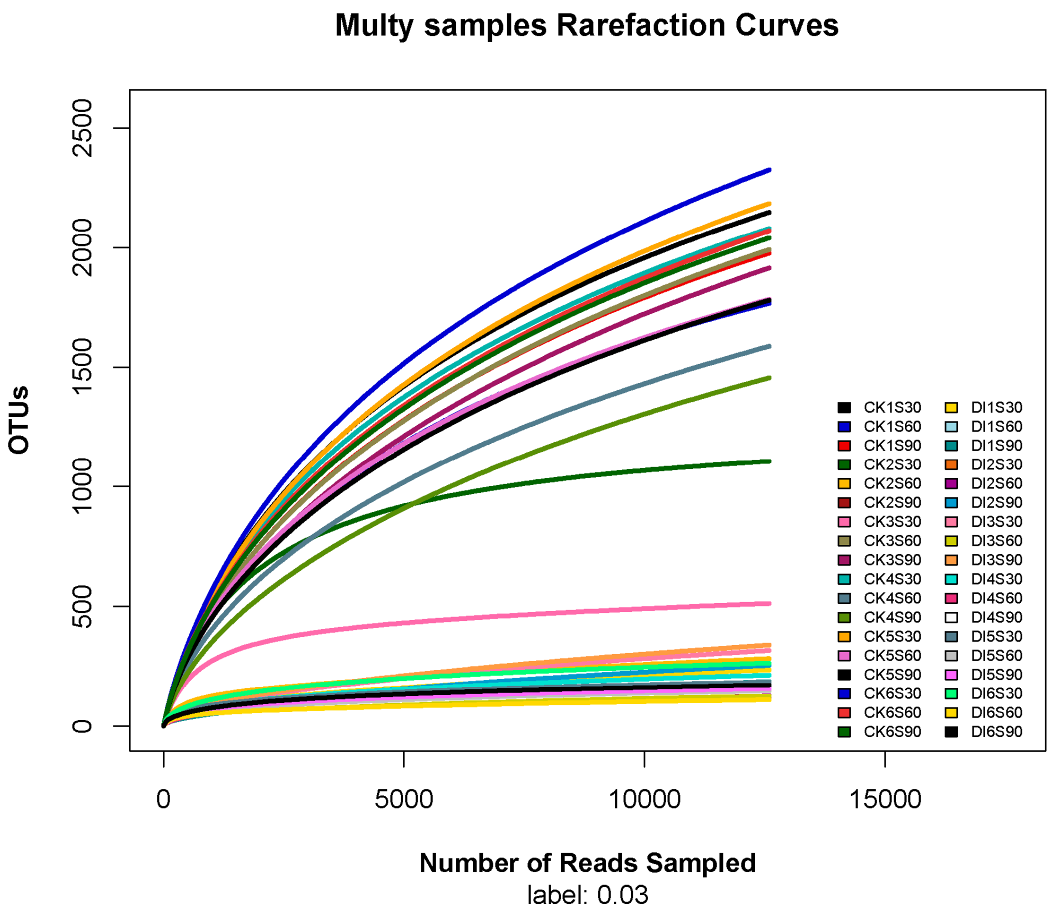

Figure S3. Species dilution curve.

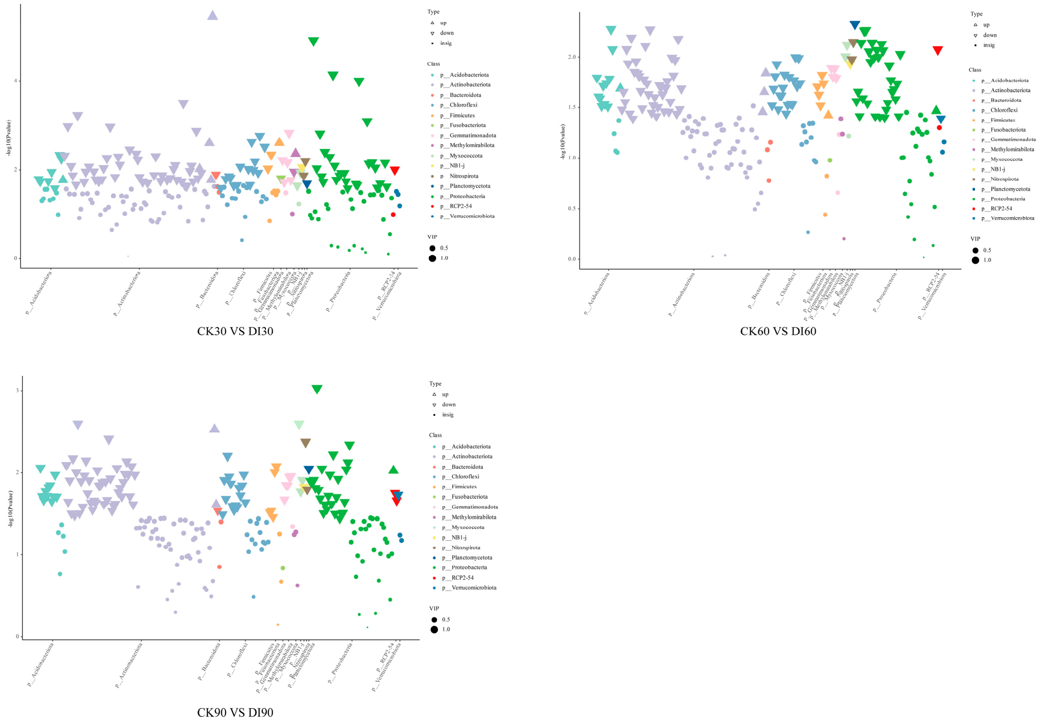

Figure S4. Community structure in treatment and control group.

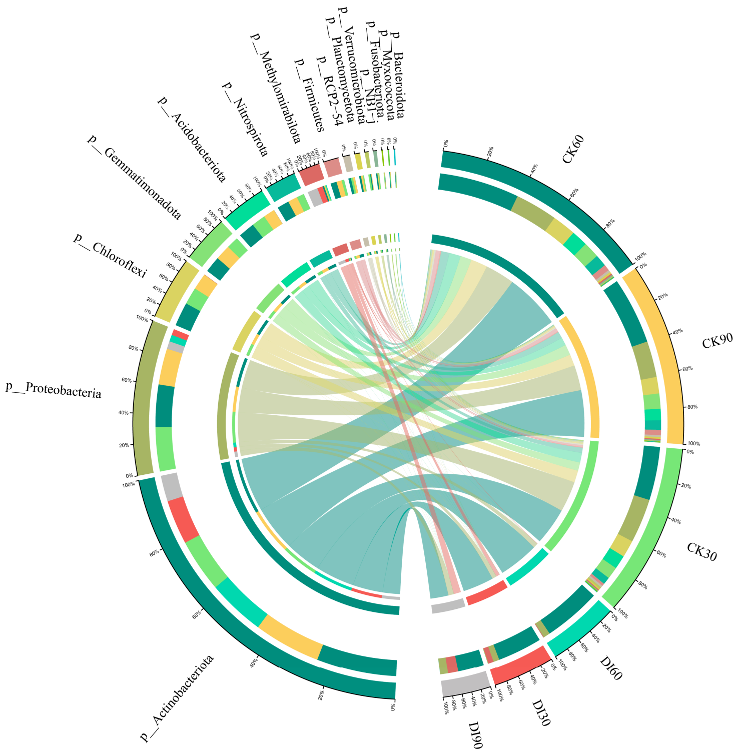

Figure S5. Circos plot at the level of the grouping genus classification.

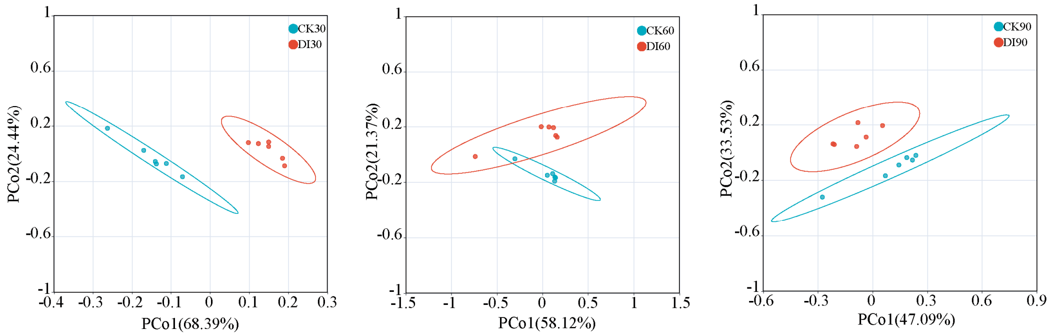

Figure S6. PcoA analysis based on PICRUSt2 functional prediction.

Table S1. Soil physical and chemical properties at different depths of the sampling point.

	AN(mg/kg)			AK(mg/kg)			AP(mg/kg)		
	30	60	90	30	60	90	30	60	90
S1	80.1	29.4	47	319.4	138.3	98.8	32.6	18.6	6.6
S2	106	102.2	32.9	168.6	98.9	74.7	12.2	7.3	5.3
S3	119	96.3	39.2	202.6	156.5	93.4	11.1	6.7	5
S4	91.1	64	20.9	180.2	95.6	72.1	13.8	7.4	6
S5	117	104.3	81.7	134.9	93.3	66.9	11.8	9.6	7.5
S6	63.1	23.4	13.8	124.9	91.8	79.0	10.0	8.4	6.7

Table S2. Characterization of microbial network interactions before and after treatment.

	CK30	CK60	CK90	DI30	DI60	DI90
Network density	0.035	0.028	0.034	0.094	0.131	0.119
Average Clustering coefficient	0.551	0.575	0.510	0.901	0.936	0.867
Perscent of positive edges	77.92	91.38	87.5	99.89	99.30	100.00
Number of negative edges	22.08	8.62	12.50	0.11	0.70	0.00
Number of total edges	865	696	824	918	142	949
Number of nodes	222	223	220	140	47	127
Modularity	0.928	0.862	0.887	0.610	0.522	0.418

Table S3. PICRUSt2 carbon and nitrogen metabolism function prediction KEGG Orthology (KO) annotation information.

Nitrogen metabolism	
Function	Description
K00259	ald; alanine dehydrogenase [EC:1.4.1.1]
K00260	gudB, rocG; glutamate dehydrogenase [EC:1.4.1.2]
K00261	GLUD1_2, gdhA; glutamate dehydrogenase (NAD(P)+) [EC:1.4.1.3]
K00262	E1.4.1.4, gdhA; glutamate dehydrogenase (NADP+) [EC:1.4.1.4]
K00265	gltB; glutamate synthase (NADPH/NADH) large chain [EC:1.4.1.13 1.4.1.14]
K00266	gltD; glutamate synthase (NADPH/NADH) small chain [EC:1.4.1.13 1.4.1.14]
K00278	nadB; L-aspartate oxidase [EC:1.4.3.16]
K00284	E1.4.7.1; glutamate synthase (ferredoxin) [EC:1.4.7.1]
K00322	sthA, udhA; NAD(P) transhydrogenase [EC:1.6.1.1]
K00360	nasB; assimilatory nitrate reductase electron transfer subunit [EC:1.7.99.-]
K00362	nirB; nitrite reductase (NADH) large subunit [EC:1.7.1.15]
K00363	nirD; nitrite reductase (NADH) small subunit [EC:1.7.1.15]
K00366	nirA; ferredoxin-nitrite reductase [EC:1.7.7.1]
K00368	nirK; nitrite reductase (NO-forming) [EC:1.7.2.1]
K00370	narG, narZ, nxrA; nitrate reductase / nitrite oxidoreductase, alpha subunit [EC:1.7.5.1 1.7.99.-]
K00371	narH, narY, nxrB; nitrate reductase / nitrite oxidoreductase, beta subunit [EC:1.7.5.1 1.7.99.-]
K00372	nasA; assimilatory nitrate reductase catalytic subunit [EC:1.7.99.-]
K00374	narI, narV; nitrate reductase gamma subunit [EC:1.7.5.1 1.7.99.-]
K00376	nosZ; nitrous-oxide reductase [EC:1.7.2.4]
K00609	pyrB, PYR2; aspartate carbamoyltransferase catalytic subunit [EC:2.1.3.2]
K00611	OTC, argF, argI; ornithine carbamoyltransferase [EC:2.1.3.3]
K01476	E3.5.3.1, rocF, arg; arginase [EC:3.5.3.1]
K01478	arcA; arginine deiminase [EC:3.5.3.6]
K01755	argH, ASL; argininosuccinate lyase [EC:4.3.2.1]
K01915	glnA, GLUL; glutamine synthetase [EC:6.3.1.2]
K01940	argG, ASS1; argininosuccinate synthase [EC:6.3.4.5]
K01955	carB, CPA2; carbamoyl-phosphate synthase large subunit [EC:6.3.5.5]
K01956	carA, CPA1; carbamoyl-phosphate synthase small subunit [EC:6.3.5.5]
K02567	napA; periplasmic nitrate reductase NapA [EC:1.7.99.-]
K02568	napB; cytochrome c-type protein NapB
K02585	nifB; nitrogen fixation protein NifB
K02586	nifD; nitrogenase molybdenum-iron protein alpha chain [EC:1.18.6.1]
K02587	nifE; nitrogenase molybdenum-cofactor synthesis protein NifE
K02588	nifH; nitrogenase iron protein NifH [EC:1.18.6.1]
K02591	nifK; nitrogenase molybdenum-iron protein beta chain [EC:1.18.6.1]
K02592	nifN; nitrogenase molybdenum-iron protein NifN

K02593	nifT; nitrogen fixation protein NifT
K02594	nifV; homocitrate synthase NifV [EC:2.3.3.14]
K03343	puo; putrescine oxidase [EC:1.4.3.10]
K04561	norB; nitric oxide reductase subunit B [EC:1.7.2.5]
K05601	hcp; hydroxylamine reductase [EC:1.7.99.1]
K10536	aguA; agmatine deiminase [EC:3.5.3.12]

Carbon metabolism	
Function	Description
K00024	mdh; malate dehydrogenase [EC:1.1.1.37]
K00027	ME2, sfcA, maeA; malate dehydrogenase (oxaloacetate-decarboxylating) [EC:1.1.1.38]
K00030	IDH3; isocitrate dehydrogenase (NAD+) [EC:1.1.1.41]
K00031	IDH1, IDH2, icd; isocitrate dehydrogenase [EC:1.1.1.42]
K00134	GAPDH, gapA; glyceraldehyde 3-phosphate dehydrogenase [EC:1.2.1.12]
K00200	fwdA, fmdA; formylmethanofuran dehydrogenase subunit A [EC:1.2.7.12]
K00201	fwdB, fmdB; formylmethanofuran dehydrogenase subunit B [EC:1.2.7.12]
K00202	fwdC, fmdC; formylmethanofuran dehydrogenase subunit C [EC:1.2.7.12]
K00672	ftr; formylmethanofuran--tetrahydromethanopterin N-formyltransferase [EC:2.3.1.101]
K00873	PK, pyk; pyruvate kinase [EC:2.7.1.40]
K00927	PGK, pgk; phosphoglycerate kinase [EC:2.7.2.3]
K01601	rbcL; ribulose-bisphosphate carboxylase large chain [EC:4.1.1.39]
K01602	rbcS; ribulose-bisphosphate carboxylase small chain [EC:4.1.1.39]
K01623	ALDO; fructose-bisphosphate aldolase, class I [EC:4.1.2.13]
K01624	FBA, fbaA; fructose-bisphosphate aldolase, class II [EC:4.1.2.13]
K01637	E4.1.3.1, aceA; isocitrate lyase [EC:4.1.3.1]
K01638	aceB, glcB; malate synthase [EC:2.3.3.9]
K01647	CS, gltA; citrate synthase [EC:2.3.3.1]
K01689	ENO, eno; enolase [EC:4.2.1.11]
K01834	PGAM, gpmA; 2,3-bisphosphoglycerate-dependent phosphoglycerate mutase [EC:5.4.2.11]
K03518	coxS; aerobic carbon-monoxide dehydrogenase small subunit [EC:1.2.5.3]
K03519	coxM, cutM; aerobic carbon-monoxide dehydrogenase medium subunit [EC:1.2.5.3]
K03520	coxL, cutL; aerobic carbon-monoxide dehydrogenase large subunit [EC:1.2.5.3]
K10713	fae; 5,6,7,8-tetrahydromethanopterin hydro-lyase [EC:4.2.1.147]
K10714	mtdB; methylene-tetrahydromethanopterin dehydrogenase [EC:1.5.1.-]
